# Amide Bonds Meet Flow Chemistry: A Journey into Methodologies and Sustainable Evolution

**DOI:** 10.1002/cssc.202102708

**Published:** 2022-02-01

**Authors:** Antonella Ilenia Alfano, Heiko Lange, Margherita Brindisi

**Affiliations:** ^1^ SPOTS-Lab – Sustainable Pharmaceutical and Organic Technology and Synthesis Laboratory University of Naples ‘Federico II', Department of Pharmacy Via Domenico Montesano 49 80131 Naples Italy; ^2^ University of Milano-Bicocca Department of Earth and Environmental Sciences Piazza della Scienza 1 20126 Milan Italy

**Keywords:** amide bonds, flow chemistry, green chemistry, peptides, sustainable synthesis

## Abstract

Formation of amide bonds is of immanent importance in organic and synthetic medicinal chemistry. Its presence in “traditional” small‐molecule active pharmaceutical ingredients, in linear or cyclic oligo‐ and polypeptidic actives, including pseudopeptides, has led to the development of dedicated synthetic approaches for the formation of amide bonds starting from, if necessary, suitably protected amino acids. While the use of solid supported reagents is common in traditional peptide synthesis, similar approaches targeting amide bond formation in continuous‐flow mode took off more significantly, after a first publication in 2006, only a couple of years ago. Most efforts rely upon the transition of traditional approaches in flow mode, or the combination of solid‐phase peptide synthesis principles with flow chemistry, and advantages are mainly seen in improving space‐time yields. This Review summarizes and compares the various approaches in terms of basic amide formation, peptide synthesis, and pseudopeptide generation, describing the technological approaches and the advantages that were generated by the specific flow approaches. A final discussion highlights potential future needs and perspectives in terms of greener and more sustainable syntheses.

## Graphical abstract

**Find your flow**: This Review summarizes and compares flow‐aided methodologies for basic amide formation, peptide synthesis, and peptidomimetic framework generation. Beyond describing the technological approaches and showcasing advantages generated by the specific flow protocols, it offers a critical point of view on future needs and perspectives towards greener and more sustainable syntheses of amide bond‐containing compounds.

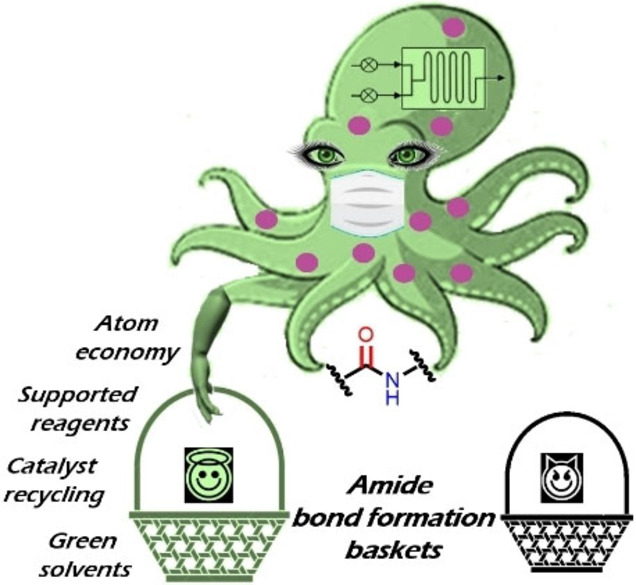

## 1 Introduction

### 1.1 Amide bonds: relevance and efforts towards sustainable procedures for their generation

The amide moiety is one of the most ubiquitous chemical bonds in nature. Peptides, proteins, clinically approved synthetic and naturally occurring drug molecules, as well as a wide variety of chemical probes contain at least one amide functionality.[^1^, ^2^, ^3^] The synthesis of amide bonds remains one of the most frequently performed reactions; accordingly, amide formation reactions represent around 16 % of the total reactions employed for the synthesis of new drugs.[^4^, ^5^]

The amide group deserves a special role among functional groups contained in marketed drugs and clinical candidates, due to its unique hydrogen bonding capability. If one considers the prevailing keto tautomer, an amide group contains two types of hydrogen bonding sites, namely the carbonyl group acting as a hydrogen bond acceptor, and the amine group behaving as hydrogen bond donor. The lone pair of the amine nitrogen is, instead, not prone to act as hydrogen bond acceptor due to its delocalization.^[3]^

A peptide bond, a term inaccurately utilized interchangeably with the term amide bond, consists of an amide composed between an α‐amino nitrogen from one amino acid and the carboxylic moiety of a second amino acid. In peptide chemistry, the partial double‐bond character of the amide group results in planar geometry, thus justifying the existence of *cis* or *trans* conformation. Nearly all proteins in their folded state adopt the *trans* configuration, which guarantees reduction of the steric hindrance between groups linked to the α‐carbon atoms.^[6]^

Despite the ubiquitous presence of amides in nature, the chemists′ toolbox for the generation of amides has remained more or less unchanged over the past decades. Synthetic methods still heavily rely on classical stoichiometric reactions between carboxylic acids and amines in the presence of diimide‐based coupling reagents or transformations of acids into more reactive species, followed by reactions with amines, which make them nonoptimal from the perspective of atom economy and green chemistry.[^7^, ^8^, ^9^]

Accordingly, the most frequently employed method for the preparation of amides involves the preliminary activation of carboxylic acid derivatives, in form of acid chlorides, anhydrides, or esters, and the subsequent reaction with amine partners. Alternatively, carboxylic acids and amines can be reacted in the presence of stoichiometric amounts of dedicated coupling reagents, such as carbodiimides or 1*H*‐benzotriazole derivatives, which guarantee in‐situ activation of the carboxylic acid.[^10^, ^11^, ^12^] The reactions between carboxylic acids or carboxylic esters and amines lead to corresponding amides only at elevated temperatures (indicatively ranging from 110 to 140 °C) or under microwave conditions in the absence of catalysts.[^13^, ^14^]

The development of novel catalytic amide bond formation reactions is among the contemporary synthetic challenges for the medicinal chemistry research.^[15]^ Additionally, in 2019, the development of safer, effective, and more sustainable methods for amide formation was included in the Ten Key Green Chemistry Research Areas.^[16]^ Therefore, both the search for innovative methods for amide bond formation as well as the implementation of technologies enabling more sustainable protocols for amide synthesis and scale‐up is a current focus for academic and industrial research groups.

Catalytic variations of direct amide bond formations between commonly available carboxylic acids, esters, or amides and amines have only recently been developed; these, however, generally require the presence of organocatalysts[^17^, ^18^, ^19^, ^20^, ^21^, ^22^, ^23^, ^24^, ^25^, ^26^] or expensive and environmentally harmful transition metal catalysts.[^1^, ^27^, ^28^, ^29^, ^30^, ^31^, ^32^, ^33^, ^34^, ^35^, ^36^]

In this context, the assessment of cheap, non‐toxic, and abundant metal salts as potential catalysts is of pivotal importance in order to reduce the environmental impact of these processes. Among them, alkali metals and alkaline earth metals possess good catalytic properties for direct transamidation of carboxylic esters.^[37]^ Recently, elegant conversions of carboxylic esters into the corresponding amides were accomplished in the presence of catalytic amounts of simple sodium methoxide or potassium *tert*‐butoxide under mild reaction conditions.[^18^, ^21^]

The use of thiocarbamates, easy to prepare and bench stable, was also disclosed. In particular, these intermediates were found to be optimum partners for Grignard reagents for the generation of hindered amides. Moreover, the use in 2‐MeTHF and the possibility of recovering the thiolate leaving group as diphenyl disulfide make the method remarkable also from the sustainability standpoint.^[38]^ Sustainability challenges for amide bond formation were also faced focusing on different coupling mediators and reaction media. Accordingly, 2,4,6‐trichloro‐1,3,5‐triazine (TCT) was employed as a convenient condensing agent in deep eutectic solvents (DES) or under mechanochemical solvent‐free conditions.[^39^, ^40^] Mechanochemical protocols were also recently coupled with the use of uronium‐based coupling reagents^[41]^ or for elegant direct transamidations of esters in the presence of calcium nitride.^[42]^ Application of photochemical processes has also been recently unveiled for a more sustainable formation of amide bonds.^[43]^

### 1.2 Flow chemical transformations

Flow chemistry is an innovative technological approach towards a greener and sustainable organic synthesis.^[44]^ Flow chemistry realizes reactions in micro‐ or meso‐scale reactors for reaction screening and route optimization, but has been optimized also on high technological levels easily capable of producing kilograms of active pharmaceutical ingredients (APIs) in a day.[^45^, ^46^, ^47^, ^48^, ^49^, ^50^, ^51^, ^52^, ^53^] Obviously, latest developments regarding the penetration of flow chemistry in production fields benefit from notable advances in adjacent technological fields, such as 3D‐printing of polymers, but also so‐called 3D‐printing of metals.[^54^, ^55^, ^56^, ^57^] At any level, benefits result from an improvement of mixing, heat and mass transfer, smaller net reaction volumes, and facilitated pressure control that lead to improvement of conversions, yields, expenditure of time, amounts of solvents needed, and so on. Furthermore, the continuous‐flow reaction has substantial advantages over the batch reaction in terms of its superior safety, and a generally improved environmental compatibility profile.

In the context of academic and earlier‐stage chemical and pharmaceutical research activities, the term “flow chemistry” is nowadays commonly connotated with small laboratory scales, in which different reactor types made from various materials, normally polytetrafluoroethylene (PTFE), polyether ether ketone (PEEK), glass, or Hastelloy, are used to usually produce gram scales of small organic molecules. Flow‐based chemical processing principally also allows for the in‐line purification of the products and the selective “catch and release” of the reaction by‐products, thus minimizing the use of solvents for purification processes.

Performing a chemical reaction in a continuous or segmented continuous‐flow chemical reactor does not mean that any given chemical transformation turns into a green and sustainable reaction. As a matter of fact, the first works detailing flow chemistry at the “common” academic lab research scale and context do not necessarily adhere to the twelve principles of Green Chemistry.[^58^, ^59^] The application of flow chemistry concept greatly facilitates, however, compliance with the ideas of green and/or sustainable chemistry. Only over time, the flow chemistry groups tried to realize the reactions putting emphasis above all on the aspects “green” and “environmentally friendly”.[^45^, ^48^, ^51^, ^60^, ^61^] The field of flow chemistry, rapidly moving since essentially two decades, is regularly described in Reviews, in which either technological aspects, dedicated reactions types, and/or realizations of important structural motifs in continuous‐flow mode are comprehensively discussed.[^62^, ^63^, ^64^, ^65^] Most interestingly and unexpectedly, to the best of our knowledge, such a dedicated Review examining amide formations in flow has not yet been presented.

Just like other research efforts involving emerging and existing technological tools, flow chemistry approaches are increasingly appearing as enabling technologies in support of more sustainable amide bond syntheses. In particular, the potential to be synergized with the use of greener solvents and reaction conditions, as well as the compatibility with supported reagents, (bio)catalysts, and scavenger resins make flow‐based approaches a valuable tool towards the sustainable generation of amide bonds.

**Figure 1:**
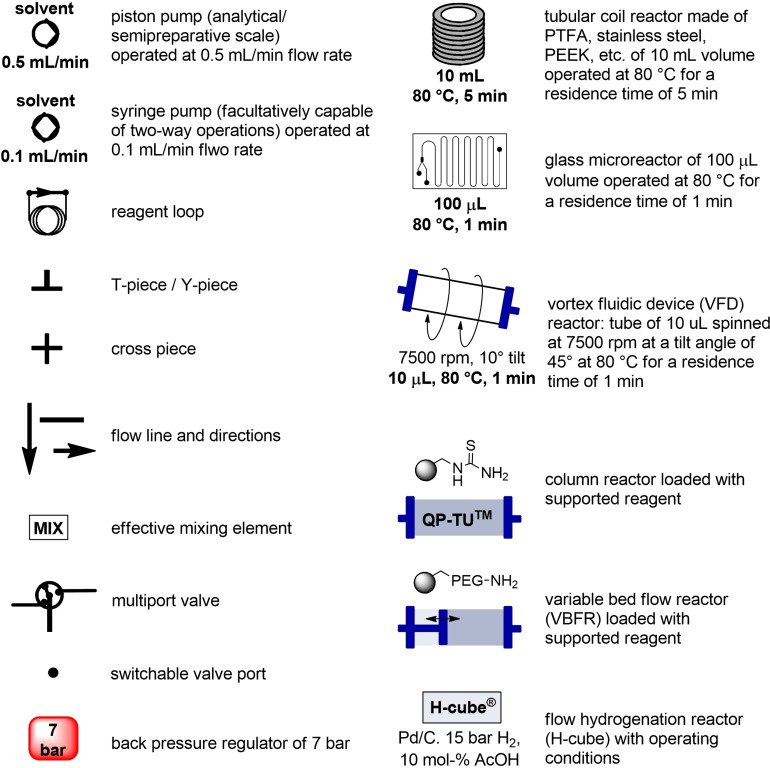
Unified symbol language used in Tables and Schemes throughout the manuscript for the most common components of flow chemistry set‐ups at laboratory scales used in the syntheses of amide bonds.

In the next paragraphs we will examine the latest progresses in the generation of amide and peptide bonds employing continuous‐flow chemistry technologies. In general, beyond showcasing and discussing flow‐based methodologies in terms of efficacy, improvement of space‐time yields, and scaling‐up potential, also green chemistry‐ and sustainability‐related aspects of the developed protocols are discussed. For describing the progresses made over the last 15 years, we use in Tables and Schemes a unified symbol language that was proposed in a previous work^[45]^ and that aims at facilitating comparison of approaches across research fields connected to, or interested in, machine‐assisted chemical synthesis, safer chemical processing, and so on. Figure 1 lists in this respect the symbols used throughout this Review on the basis of our symbol definitions introduced recently.^[45]^

## 2 Discussion

### 2.1 Synthesis of amide bonds in continuous flow

**Scheme 1:**
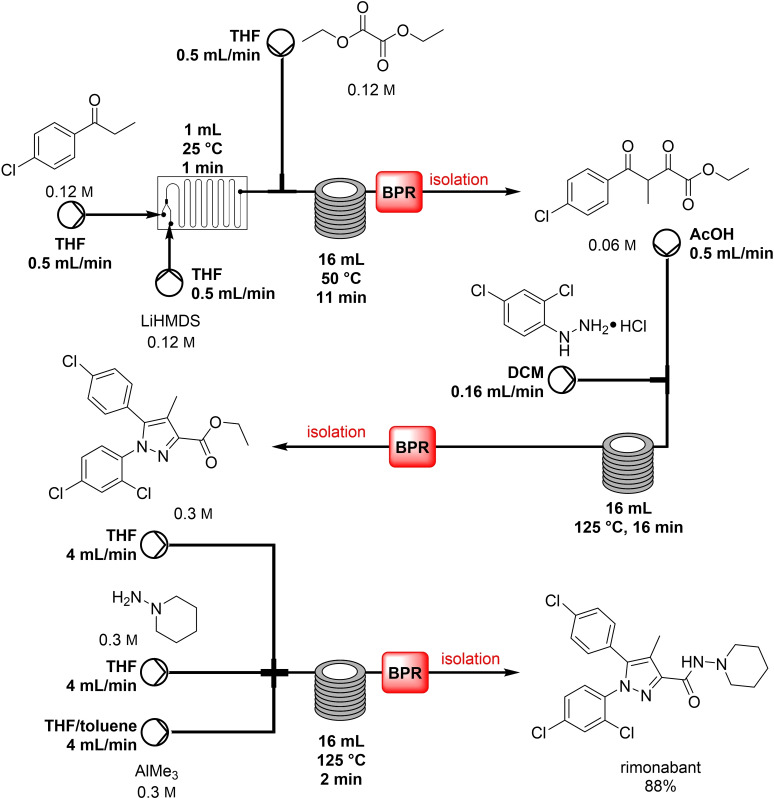
Flow synthesis of rimonabant by Seeberger and co‐workers with TMA‐triggered hydrazide formation as concluding step.^[66]^ Back pressure values have not been detailed.

One of the first efforts concerning the flow synthesis of amide bond dates back only to 2008 and was realised by Seeberger and co‐workers, who reported a microreactor‐based, trimethylaluminum (TMA)‐mediated amide bond formation and its application to the synthesis of two drugs: rimonabant and efaproxiral (Table 1, entry 1; Scheme 1).^[66]^ TMA is the most reactive and volatile among all organoaluminum compounds and extremely pyrophoric. Using a flow chemistry protocol was thus seen as a means to overcome the instability issues of aluminum–amide intermediate at elevated temperatures. On an 8.0 mmol scale, 0.3 m solutions in THF of ester, amine, and TMA were mixed equimolar in a simple mixer and passed through a 16 mL tube reactor at a total flow rate of 8 mL min^−1^ at 125 °C. The reaction solution was collected in a mixture of EtOAc and HCl (aq. 3 %) and purified on silica gel. In only 2 min, an amide moiety was achieved. The reactions were typically performed on 8 mmol scale; however, they could be scaled to 0.1–0.2 mol by running them continuously. A library of 17 compounds, with aliphatic and aromatic esters and amines, was synthesized with moderate to excellent yields that were found often to be higher than those obtained in corresponding microwave reactions. Moreover, rimonabant and efaproxiral were synthesized as exemplary amide‐containing drugs, demonstrating the efficiency of the process in real cases.

**Table 1:** Amide bond formation in segmented continuous flow.

Entry	Starting compound(s)	Coupling reagent(s)/ catalysts	Targeted structural scope	Solvent/ *T*/*p*	Reactor	# Examples/ yields [%]	Ref.
1	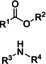	TMA	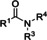	THF 125 °C	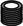	17 65–96	[66]
2	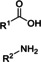	–	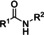	neat 245–280 °C 25–35 bar	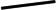	2 93–94	[67]
					of γ‐Al_2_O_3_, MW		
3	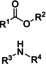	LHMDS	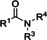	THF 25 °C	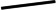	25 24–100	[68]
4	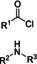	TEA	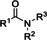	CHCl_3_ 25 °C	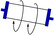	28 49–99	[69]
5	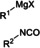	CuBr_2_ (cat.)	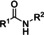	THF 25 °C	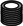	32 45–98	[70]
6	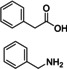	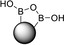	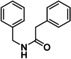	toluene 110 °C	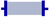	1 17^[a]^	[71]
7	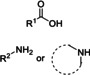	CS_2_, DMAP, Al_2_O_3_	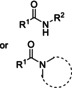	MeCN 200 °C	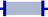	9 primary+6 secondary 94–98	[72]
8	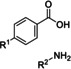	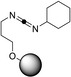	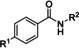	DCM–DMF 60 °C	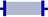	16 76–99^[a]^	[73]
9	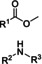	ZrO_2_	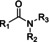	diglyme 160 °C	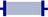	26 19–99	[74]
10	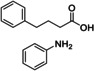	TiO_2_/NiFe_2_O_4_	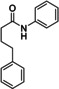	*p*‐xylene 150 °C	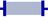	1 0.02 mol g_TiO2_^−1^ s^−1^ production rate	[75]
					with inductive heating		
11	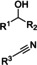	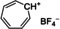	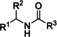	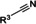	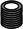	7 82–93	[76]
				150 °C			

[a] Only conversions reported.

Four years later, in 2012, Morschhäuser et al. presented a microwave‐assisted continuous‐flow synthesis of amides on industrial scale (Table 1, entry 2).^[67]^ The synthesis uses equimolar amounts of unprotected carboxylic acids and amines to form amides at high temperatures and high pressures of up to 35 bar, pumping in fact a solution of the initially formed ammonium salt through the reactor. Tubing was realized from microwave‐transparent γ‐Al_2_O_3_. Two examples were realized, at flow rates of 3.5 and 5.6 L h^−1^, respectively. Reported yields were very high. The authors claim that the whole process was also very energy efficient. The concept could be extended for realizing esters and benzimidazoles.

In 2014, Alcázar and co‐workers presented a greener protocol for the direct aminolysis of esters with amines, using lithium bis(trimethylsilyl)amide (LHMDS) as strong non‐nucleophilic base (Table 1, entry 3).^[68]^ Starting from ethyl benzoate and 4‐amino‐pyridine, an optimization of equivalents, time, and temperature led to the winning protocol: two solutions, ester and amine at 0.45 and 0.5 m, respectively, in dry dimethylformamide (DMF) on the one hand, and LHMDS, 1 m in dry THF, were both pumped at a rate of 0.25 mL min^−1^ using syringe pumps into a mixing element. The mixed solutions were passed in a 1 mL mixing chip held at 25 °C for 2 min of residence time, after which the reaction was quenched off‐line in an NH_4_Cl solution. A library of 25 compounds was synthesized to explore the scope of this approach, including different alkyl and aryl esters, alkyl and aryl amines, and various functional groups present in both building blocks; adapting the reaction conditions for each compound, yields between 24 and 100 % were obtained. Furthermore, a telescoped approach combined this procedure with a previous work of the same group based on the flow carbonylation of bromobenzene with 2,4,6‐trichlorophenyl formate as CO source without the need of an external gas cylinder, thus furnishing *N*‐(pyridin‐4‐yl)benzamide in 65 % yield.^[77]^

**Scheme 2:**
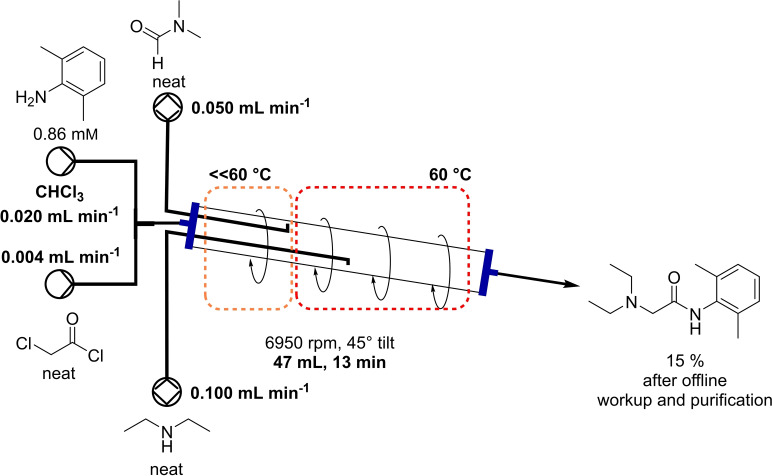
Flow synthesis of lidocaine by Raston and co‐workers.^[69]^

**Scheme 3:**
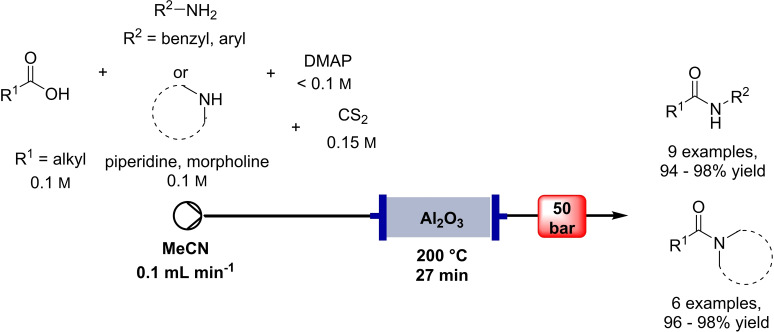
Flow‐based amide synthesis using Al_2_O_3_ as Lewis acid catalyst by Orsy et al.^[72]^

Raston and co‐workers, in 2015, reported a continuous‐flow approach for the amide synthesis using a novel thin film vortex fluidic device (VFD) (Table 1, entry 4; Scheme 3).^[69]^ The reagents were introduced into the inclined rapidly rotating tube and proceeded up to the walls of the tube for exiting. The short reaction time of 80 s increased the yield as compared to the conventional batch mode. A solution of 8.61 mmol of amine and 10.8 mmol triethylamine in 10 mL CHCl_3_ was added to the flow reaction by means of a first syringe pump and were mixed with 1.75 equiv. acyl chloride in 10 mL CHCl_3_, furnished by a second syringe pump. The VFD was set at a tilt angle of 45° relative to the horizontal position, for a rotational speed of 6950 rpm. The amide bond was formed with no complex post‐VFD operations required; the product was simply collected, washed with 2 m HCl, dried, and recrystallized for purification. A library of 28 products was obtained with yields between 49 and 99 %, exceeding batch yields. Batch‐type reactions typically suffer from violent exothermic behaviors that cause reagent degradation, hence lower yields, and safety concerns. The authors applied this method to the first total synthesis of lidocaine in flow, compartmentalizing a single VFD by positioning the capillary feeds strategically along the tube and using localized heating (Scheme 2).

Another three years later, in 2018, Williams et al. reported a convenient amide synthesis starting from isocyanates and Grignard reagents (Table 1, entry 5).^[70]^ In a simple set‐up, they mixed a stream of Grignard reagent in THF with a stream of an equimolar amount of isocyanate in THF, containing also the catalytic amount of copper(II) bromide, in a T‐piece at respective flow rates of each 5 mL min^−1^. After a very short residence time of approximately 11 s, the product was collected for off‐line work‐up in an aqueous solution of NH_4_Cl. Using this approach, a library of 32 compounds was realized, with isolated yields up to 98 %.

In 2019, Whiting and co‐workers published a paper on the formation of various amides using a polystyrene‐supported boronic acid catalyst (Table 1, entry 6).^[71]^ This catalyst is readily prepared by heating a mixture of 4‐styreneboronic acid, styrene, 1,4‐divinylbenzene, and azobisisobutyronitrile (AIBN) in 1‐dodecanol at 85 °C; originally produced in monolithic form, the authors grinded the block for arriving at a powdered form that can still be easily recovered by simple filtration. After demonstrating that this catalyst, exhibiting 5 % active loading, stays active across five cycles during the generation of a library of 20 compounds in batch, the reaction was transferred into a flow system in form of a packed‐bed reactor method using an Omnifit glass column filled with a mixture of the powdered polymeric catalyst and celite, and the reactor was washed with toluene at 0.100 mL min^−1^ for 1 h before heating up. For amide formation, an equimolar, approximately 0.6 m solution of carboxylic acid and amine in toluene was then pumped through the column reactor heated to 130 °C at a flow rate of 0.100 mL min^−1^. In total, the set‐up produced 1.80 g of amide, corresponding to 17 % conversion, over 116 h at a 15.5 mg h^−1^ rate, with activity of the catalyst lasting during this operational period. The catalytic activity was maintained for over 4.5 days of continuous operation.

Orsy et al. presented in 2020 a catalytic route to amides using carbon disulfide and alumina as activation agent and catalyst, respectively (Table 1, entry 7; Scheme 3).^[72]^ Starting from benzylamine and 4‐phenylbutyric acid as substrates dissolved to 100 mm in MeCN, the reaction was carried out in a home‐made flow reactor consisting of a stainless‐steel preheating coil and a stainless‐steel high‐pressure liquid chromatography (HPLC) column as catalyst housing. Optimized reaction conditions required 1.5 equiv. of CS_2_, catalytic amounts of 4‐(dimethylamino) pyridine (DMAP), and an excess of Al_2_O_3_ as sustainable Lewis acid catalyst; MeCN as a less problematic dipolar aprotic solvent was used, and the reactor was run at 200 °C, 50 bar, and 0.1 mL min^−1^ flow rate. The optimized protocol worked for the combination of three different carboxylic acids with five different amines to give a library of 15 amides of unspecified purity with yields between 94 and 98 % after filtration through a short pad of silica gel. The scale‐up potential of the flow process was demonstrated synthesizing 2 and 10 g of the product after around 13 h and 3 days of operation, respectively, without a significant loss of productivity of the system.

**Scheme 4:**
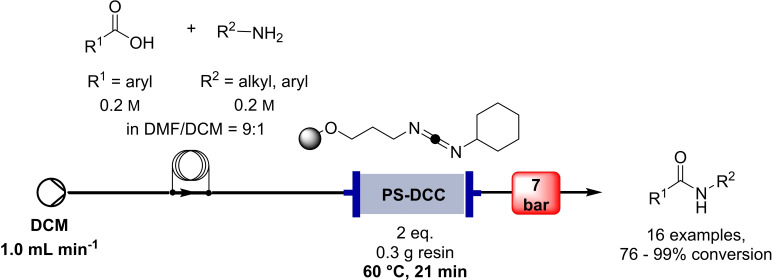
Flow‐based amide synthesis on polymer‐supported DCC developed by Gordon and co‐workers.^[73]^

In the same year, Gordon and co‐workers developed a simple flow protocol using polymer‐bound carbodiimide as activating agent for reacting benzoic acids with various amines (Table 1, entry 8; Scheme 4).^[73]^ They tested different polymer‐bound coupling reagents, and *N*,*N*’‐dicyclohexylcarbodiimide (DCC) derivative PS‐*N*‐(3‐(benzyloxy)propyl)‐*N*‐cyclohexylmethanediimine turned out to give the best results. A glass column reactor was loaded with 400 mg of this coupling resin (i. e., 0.536 mmol or 2.0 equiv.), through which was passed a continuous stream of dichloromethane (DCM) at 2 mL min^−1^ for achieving initial catalyst swelling. Upon complete resin swelling, the column was heated to 60 °C. The starting materials (Cbz‐β‐alanine and benzylamine) were mixed off‐line in equimolar amounts in form of 0.27 m solution in each component in DCM containing 10 % DMF; this solution was brought to reaction after being injected in a segmented continuous flow mode. In around 90 s retention time, 16 amides were so reached with conversions between 76 and 99 %, using only a single batch of resin. This method can be applied also to provide dipeptides and cyclic peptides (see below).

In 2021, a green, fast, sustainable, and metal‐free amidation in continuous flow was developed by Kobayashi and co‐workers, starting from unactivated esters and amines using a heterogeneous ZrO_2_ catalyst (Table 1, entry 9).^[74]^ A mixed solution of methyl benzoate and a 1.2‐fold excess of *n*‐hexylamine in diglyme was pumped at 0.1 mL min^−1^ through a column filled with an excess amount of polymer‐supported catalyst and held at 160 °C. The catalyst was prepared via a wet impregnation method from an aqueous solution of zirconium oxychloride hydrate, ZrCl_2_O ⋅ 8 H_2_O. For a 6 g catalyst‐loaded column the maximum concentration loading would be 0.4 m of ester. The desired amide was obtained in isolated yield of 98 %. To test the durability of the catalyst, the flow set‐up was used to produce various batches of the amide in yields varying between 94 and 98 % over a 140 h period. The observable gradual decrease in yield after prolonged usage times of the catalyst was attributed to the accumulation of a small amount of reactant or product on the catalyst surface. A library of 26 products was achieved with isolated yields of 19–99 %.

Also very recently, in 2021, Liu and Rebrov developed another atom‐efficient way to amide bonds through the use of titania‐based catalyst in form of a magnetic composite (Table 1, entry 10).^[75]^ A mixture of NiFe_2_O_4_ and sulfated titania was mechanochemically reacted in order to prepare the composite magnetic catalyst. The catalyst was used in the reaction between aniline and 4‐phenylbutyric acid (PBA) in batch as well as in a continuous‐flow reactor under both conventional and inductive heating. In flow mode, a solution of 0.1 m aniline and a 0.1 m solution of 4‐phenylbutyric acid, both in *p*‐xylene, were fed by means of HPLC pumps into the column reactor that contained the catalyst; the system was kept under 7 bar of pressure using a backpressure regulator. The catalyst was heated by an 8‐turn induction coil with a length of 70 mm. The authors emphasize that, to avoid non‐uniform heating, the conductive thermal resistance of the catalyst preparation should be much smaller than the combined thermal resistance of the reactor wall, insulation, and natural convection. In batch it was found that the reaction rate under inductive heating increased by 25 % compared with conventional heating at the same temperature (i. e., 150 °C). The catalyst could be recovered after a treatment with an air flow at 400 °C.

The latest work at the time this Review was compiled was performed by Nguyen and co‐workers (Table 1, entry 11).^[76]^ They reported a new batch method for the amide bond formation, using tropylium salts to promote the Ritter reaction.^[78]^ To demonstrate the applicability of this method, they carried out large‐scale syntheses in a continuous‐flow mode, using the nitrogen component in form of nitriles as solvent. A 0.2 m solution of an alcohol spiked with 1 mol% of tropylium tetrafluoroborate in functionalized nitrile was passed into a PTFE reactor coil heated to 150 °C at a flow rate of 0.2 mL min^−1^, for creating a total residence time of 50 min. The yields of the 7 target products obtained in flow on 20–100 mmol scale were between 82–93 %, and the authors assumed them to be comparable to or even higher than those achieved in corresponding batch processes, with purities >90 % after a simple liquid–liquid extraction off‐line to also remove the catalyst.

### 2.2 Importance of sustainable synthesis in peptide chemistry

Peptides are an incredibly important class of therapeutic agents, with more than 60 peptide drugs on the market and over 350 in clinical and preclinical development.^[79]^ Despite this undeniable primacy as therapeutically relevant agents, current methodologies for peptide synthesis still rely upon traditional protocols involving the use of hazardous reagents and solvents, with only few sparks towards a greener transition.^[80]^

The iterative assembly of peptides through solid‐phase peptide synthesis (SPPS) is among the most inefficient chemical processes, also featuring an exceptionally poor atom economy profile. The waste production associated to peptide generation is quite impressive, since an estimated five metric tons of waste are produced for every kilogram of peptide delivered.^[81]^ Therefore, there is a critical need to implement more sustainable protocols and technologies to access this relevant class of molecules.

In 2016, the American Chemical Society‐Green Chemistry Institute (ACS‐GCI) Pharmaceutical Roundtable identified the development of greener peptide processes as a critical unmet need. Among the main issues associated to sustainability of peptide synthesis protocols there is the significant amount of waste streams deriving from the use of hazardous solvents, such as *N,N*‐dimethylformamide (DMF) and *N*‐methyl‐2‐pyrrolidone (NMP), used in SPPS. In recent years, a small number of greener solvents have been screened as alternatives, such as 2‐MeTHF, cyclopentyl methyl ether, γ‐valerolactone,[^82^, ^83^] and *N*‐butylpyrrolidinone (NBP).^[84]^ However, this field still lacks breakthroughs with tangible utility and potential to replace traditional SPPS decades‐old practices. Also contributing to the poor environmental profile is the widespread employment of chromatographic techniques to release peptide products with high purity standards, which means a huge amount of solvents dedicated to this use.

The greening of the SPPS process has seen some improvements lately in chain assembly, resin cleavage, side chain deprotection, peptide work up, and the recycling of SPPS materials.^[85]^

Regarding coupling agents, current protocols mostly envisage the use of benzotriazole derivatives, in form of their uronium/aminium and phosphonium salts.[^86^, ^87^] Although these reagents are highly stable and provide high coupling efficiency and low amino acid racemization, the presence of the benzotriazole motif makes them potentially explosive.^[88]^ In addition, these reagents display very poor atom economy. New coupling reagents constantly appear in the scientific literature that are claimed to display more sustainable features. Ethyl cyano(hydroxyimino)acetate “oxyma pure”,^[89]^ used in combination with a carbodiimide, and its derived uronium salt, (1‐cyano‐2‐ethoxy‐2‐oxoethylidenaminooxy)dimethylamino‐morpholino‐carbenium hexafluorophosphate (COMU),^[90]^ generally show improved coupling efficiencies and reduced racemization chance than their benzotriazole counterparts. Also, COMU is compatible with the use of γ‐valerolactone as the solvent system, which makes it attractive from a sustainability standpoint.^[83]^

The SPPS concept first applied by Merrifield et al. set the bases for automation and iterative coupling methodologies, especially useful for the synthesis of challenging peptides.^[91]^ However, many efforts are still needed for SPPS optimization, particularly in terms of sustainability. Currently employed protocols encompass the use of large amino acid, base, and coupling agent excess; also, the commonly used fluorenylmethyloxycarbonyl (Fmoc)‐capped amino acids confer scarce atom economy and sustainability to the overall process, which also takes into consideration the large excess of piperidine necessary for removal of Fmoc moiety. An often‐overlooked waste product of SPPS is the resin itself. After a successful peptide synthesis, the peptide is released from the solid support, and the spent resin is typically disposed of in a mixed solid waste stream. Therefore, more efforts should also be devoted on developing novel methods for resin recycling, ideally universal and possibly independent of the resin type and linker used.

As mentioned above, flow chemistry possesses the credentials to boost the sustainability profile of chemical processes due to its intrinsic features, namely fast heat transfer as well as reduced energy usage and reaction times. In this context, it is nice to recall one that of the first applications of flow chemistry to SPPS dates from 1981. A system was described for solid‐phase synthesis of peptides under continuous‐flow conditions with liquid chromatographic equipment and conventional polystyrene supports. The model tetrapeptide L‐A‐G‐V was generated in 99.3 % purity in about 4 h on microporous copoly(styrene‐1 % divinylbenzene). During coupling, the preformed symmetric anhydrides were conserved by being recycled.^[92]^

The recent rebirth in flow‐based SPPS protocols took advantage from the implementation of packed‐bed flow SPPS and full system automation.[^93^, ^94^, ^95^, ^96^] A limited number of reports, however, is available for scalable continuous‐flow SPPS.[^97^, ^98^] More efforts have been performed in the context of fast, safer, and more sustainable small peptide motifs, also involving the use of *N*‐carboxyanhydrides and derivatives as an efficient alternative for amino acid activation.[^98^, ^99^] This field, nevertheless, clearly still needs substantial optimization efforts in order to provide robust alternative approaches and technologies that could ultimately lead to overall benefits in terms of efficiency and reduction of the environmental impact for peptide synthesis and production.

### 2.3 Synthesis of peptides in continuous flow

In 2006, Baxendale et al. reported one of the very first flow protocols for the multistep assembly of di‐ and tri‐peptides, using columns packed with various resins serving as reagents, catalysts, and scavengers (Table 2, entry 1).^[100]^ Two different methods were developed for the synthesis of Boc‐ or Cbz‐ and Fmoc‐protected amino acids: 4.0 equiv. of *tert*‐butyloxycarbonyl (Boc)‐ or benzyloxycarbonyl (Cbz)‐protected amino acid, 4.2 equiv. (benzotriazol‐1‐yloxy)tripyrrolidinophosphonium hexafluorophosphate (PyBoP), and 6.0 equiv. *N*,*N*‐diisopropylethylamine (DIPEA) in DMF were passed through a glass column containing 1.0 equiv. of polymer‐supported hydroxybenzotriazole (PS‐HOBt). This allowed the PS‐HOBt to catch the amino acid in its activated form for later use. After loading, two other separate glass columns, containing 1.5 equiv. of polymer‐supported 4‐(dimethylamino) pyridine (PS‐DMAP) and 1.5 equiv. TsOH on macroporous polystyrene resin (MP) support, were added in‐line the flow system, before a solution containing the second amino acid in the form of its HCl salt was passed through the three columns in series, i. e., PS‐DMAP, PS‐HOBt, and MP‐TsOH, at a flow rate of 100 μL min^−1^ for a total period of 2 h. The crude dipeptide did not require further purification. A library of 9 compounds with yields between 61 and 83 % was provided.

**Table 2:** Di‐ and tripeptide formation in segmented continuous flow.

Entry	Starting compound(s)	Coupling reagent(s)/ catalysts	Targeted structural scope	Solvent/ *T*	Reactor	# Examples/ yields [%]	Ref.
1	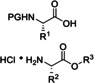	DIPEA 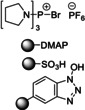	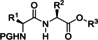	DMF 25 °C	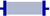	9 61–83	[100]
2	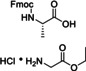	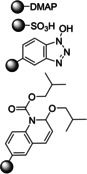	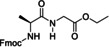	DMF 60 °C	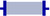	1 71	[23]
3	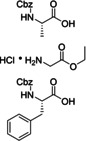	DIPEA 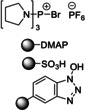	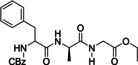	DMF 60 °C	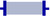	1 59	[33]
					H‐cube		
4	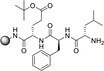	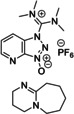	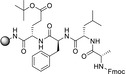	DMF 70 °C	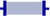	20 99^[a]^	[101]
		DIPEA					
5	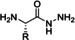	DIPEA, 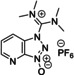	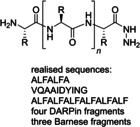	DMF 90 °C	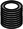	3 n.d.	[102,103]
6	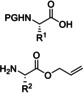	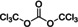 DIPEA	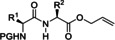	DMF–MeCN 20 °C	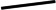	7 80–100	[104]
7	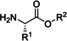	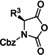	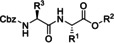	DMF 25 °C	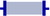	7 44–95	[105]
					H‐cube		
8	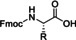	PS‐rink amide, DIPEA, 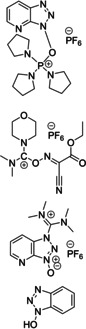	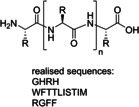	DMF 90 °C	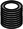	5 n.d.	[106]
9	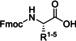	Tentagel resin, DIPEA, 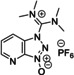	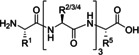	DMF 50 °C	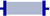	7 n.d.	[107]
10	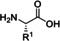	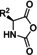	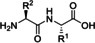	H_2_O 0–2 °C	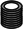	6 60–85	[108]
11	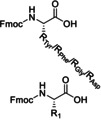	PS‐rink amide, DIPEA,	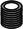	DMF 40 °C	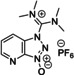	5 n.d.	[109]
		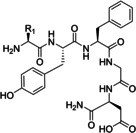					
12	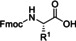	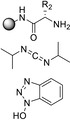	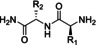	DMF 80 °C	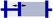	4 73–85	[110]
13	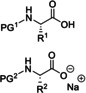	DIPEA, 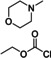	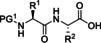	MeCN 20 °C	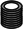	14 60–96	[111]
14	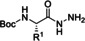	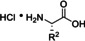	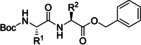	toluene–H_2_O 0 °C	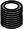	dimers: 5 70–77 trimers: 1 94	[112]
		HNO_2_, TEA					
15	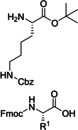	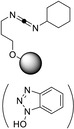	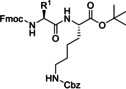	DCM–DMF 60 °C	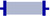	2 95–97^[a]^	[73]
		(DIPEA)					

[a] Only conversions reported.

In this set‐up, Fmoc‐protected amino acids caused low yields and unacceptable purities, most likely as a result of the DIPEA causing Fmoc deprotection. Therefore, a second protocol was designed (Table 2, entry 2), using a column packed with 1‐isobutoxycarbonyl‐2‐isobutoxy‐1,2‐dihydroquinoline on polymer support (PS‐IIDQ) to create an anhydride of the Fmoc protected amino acid, which was then passed into a column containing the PS‐HOBt. The dipeptide Fmoc‐A−G−OEt was produced in a 71 % isolated yield.

**Scheme 5:**
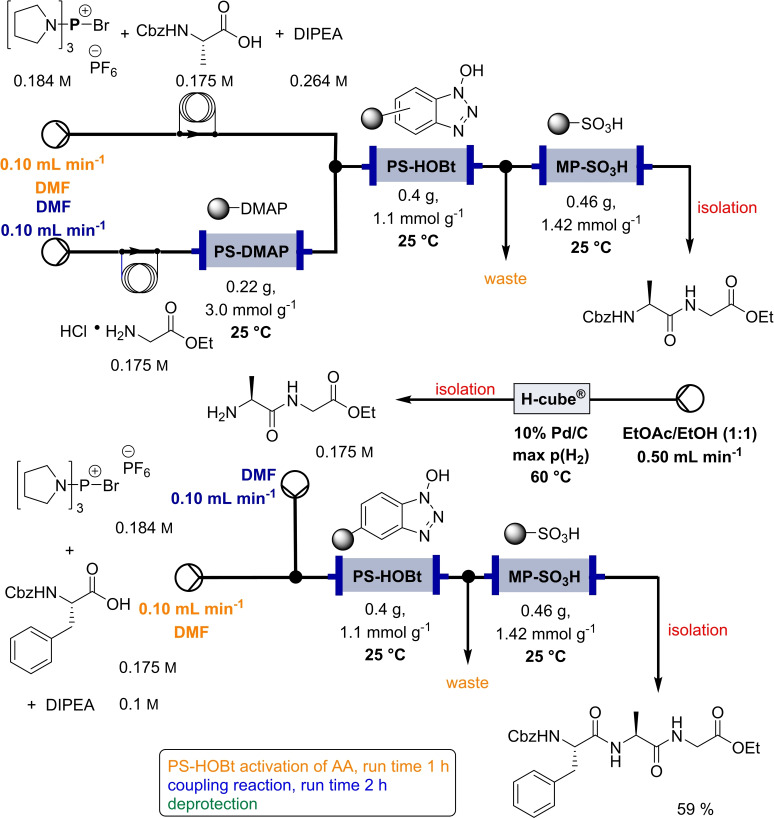
Synthesis of tripeptides on solid support in flow by Baxendale et al.^[100]^

Finally, a method for synthesizing tripeptide Cbz−F−A−G−OEt was developed with the only addition of a deprotection step through the use of an in‐line flow hydrogenation using the H‐Cube (Table 2, entry 3; Scheme 5). The desired tripeptide was isolated in 59 % yield as a single diastereoisomer and in 95 % purity. With respect to a classical batch peptide synthesis that required around 24 h, the flow process was completed in 3–4 h for dipeptide and 6–7 h for tripeptide synthesis.

A flow peptide synthesis on solid phase with reduction of amino acid excess was published in 2014 by Fülöp and co‐workers (Table 2, entry 4).^[101]^ An optimization of flow process parameters revealed that the coupling between 1.5 equiv. of Fmoc‐protected alanine and 0.03 mmol (1.0 equiv.) of leucinyl‐phenylalaninyl‐ glutamic acid [H−L−F−E(O*t*Bu)] as a Tentagel resin‐bound species required 1.5 equiv. of the standard coupling reagent *O*‐(7‐azabenzotriazol‐1‐yl)‐1,1,3,3‐tetramethyluronium hexafluorophosphate (HATU) in 1 mL of DMF at 60 bar and 70 °C and 0.15 mL min^−1^ flow rate. The coupling mixture was prepared just before the coupling reaction. For in‐line deprotection, 1 mL of 2 % (*w*/*w*) 1,8‐diazabicyclo[5.4.0]undec‐7‐ene (DBU) and 2 % (*w*/*w*) piperidine in DMF have been used. Between two chemical steps, surplus reagents and so on were washed out from the columns and the system using DMF at a flow rate of 0.15 mL min^−1^ for 5 min. With respect to scope, all of the 20 proteinogenic amino acids were coupled with conversion rates of >99 %. To demonstrate the efficiency of the methodology, also more complex sequences were synthetized, such as the acyl carrier protein 65–74 fragments H−V−Q−A−A−I−D−Y−I−N−G−NH_2_ and H−G−L−I−T−V−S−V−A−V−NH_2_. The purities of the resulting crude peptides are comparable with results reported in literature for batch processes; the flow methodology, however, requires 70 % less amino acid and 25 % less solvent. Furthermore, β‐peptide foldamers with alicyclic side chains are accessible via the proposed route in astonishing yields.^[113]^

In 2014, Pentelute and co‐workers reported a rapid flow‐based SPPS that enables the incorporation of an amino acid residue every 1.8 min under automated control or every 3 min under manual control (Table 2, entry 5).^[102]^ Coupling was performed at 60 °C by delivering a solution consisting of 2 mmol of *N*‐α‐Fmoc and side chain protected amino acid, 2 mmol *N,N,N’,N*’‐tetramethyl‐*O*‐(1*H*‐benzotriazol‐1‐yl)uronium hexafluorophosphate (HBTU), and 3 mmol DIPEA in 5 mL of DMF, at a flow are of 12 mL min^−1^, for approximately 30 s. Subsequently, coupling reagents were removed with 20 mL of DMF, delivered at 10 mL min^−1^ flow rate of 2 min, before the *N*‐α‐Fmoc protecting group was removed with 3.3 mL of 50 % (*v*/*v*) piperidine in DMF, delivered at 10 mL min^−1^ over 20 s. Excess piperidine and piperidine–DMF were removed with 20 mL of DMF delivered at 10 mL min^−1^ over 2 min to complete one coupling cycle. All peptides were synthesized on 100 mg of 1 % divinyl benzene‐crosslinked polystyrene resin. Furthermore, a scale‐up is demonstrated increasing the diameter of the vessel and flow rates to maintain residence and reaction times. To prove the utility of this system, it was applied to the synthesis of a 58‐residue tri‐helical protein based on the Z domain of protein A. (i. e., the “affibody”). With the reported system, wash times are significantly reduced from several minutes to 1 min and less.

In the same year, a flow‐based transformation was used by the same group using the same Fmoc‐based coupling chemistries as described above for the total synthesis of two proteins: a 130‐residue designed ankyrin repeat protein (DARPin) pE59 and the 113‐residue RNAse from *Bacillus amyloliquefaciens* (Barnase) using a novel fast flow peptide synthesizer (Table 2, entry 5).^[103]^ They assembled around 30‐residue polypeptides in hours rather than days with excellent crude quality and coupled approximately 1000 amino acids in around 60 h of linear synthesis time, instead of around 1000 h needed in classic synthesis. The described synthesis resulted in microgram quantities of target polypeptides; structural verification was done using mass analysis on non‐purified samples.

**Scheme 6:**
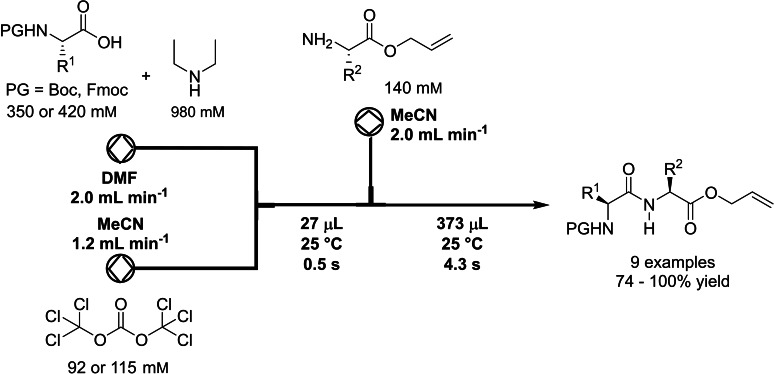
Flow amide formation using phosgene‐activation of carboxylic acids by Fuse et al.^[104]^

In 2014, Fuse et al. developed a flow route to create amide bonds, starting from various carboxylic acids, amines, and triphosgene as activator of the acids (Table 2, entry 6; Scheme 6).^[104]^ A solution of 2.5 equiv. of α‐chiral carboxylic acid in DMF was added with a syringe pump in the first mixer and reacted with phosgene, generated in situ by the reaction between 3.0 equiv. DIPEA in MeCN, and 4.0 equiv. of less toxic triphosgene in MeCN, at 20 °C. In only 0.5 s, the carboxylic acid undergoes activation in form of the corresponding acid chloride. The very reduced reaction time effectively suppressed racemization (≈3 %). A solution of 1.0 equiv. of amine in MeCN was introduced in a second mixer with syringe pump and was mixed with acid chloride at 20 °C for a residence time of 4.3 s to achieve amide bond formation. The mixture was quenched off‐line by means of an aqueous solution of NH_4_Cl and CHCl_3_. A total of 7 examples was realized, with yields between 80 and 100 %, all higher than the respective yield in batch, in only 5 s at ambient temperature. This process can be applied to dipeptide and tetrapeptide synthesis: they synthesized a tetrapeptide moiety of the depsipeptidic natural product auliride. The desired tetrapeptide, i. e., allyl *N*‐(*N‐*Fmoc‐l‐valyl)‐*N*‐methyl‐d‐leucyl)‐*N*‐methylglycyl‐l‐valinate was obtained in a good 2‐step yield of 60 % without epimerization. The same protocol could be used for the synthesis of peptidomimetics (see below).

Also in 2014, Ley and co‐workers presented a new method for the automated synthesis of peptides in flow using pre‐activated building blocks in the form of *N*‐Cbz‐protected *N*‐carboxyanhydrides (Cbz‐NCAs) (Table 2, entry 7).^[105]^ Repetitive procedures of coupling, deprotection, isolation, and purification of intermediates as needed in case of classic polypeptide synthesis were thought to be eliminated using an automated flow sequence. To start in batch, the free amino acid (AA) was activated with triphosgene in THF followed by protection with Cbz chloride, to give a stable and crystalline Cbz‐AA‐NCA as building block. Turning into flow then, l‐phenylalanine methyl ester (l‐F‐OMe, 0.10 mmol) was dissolved in 1.0 mL of DMF in a sample vial that was placed in the liquid handler at the mixing location and was mixed with a three‐fold excess of Cbz‐l‐V‐NCA in 1.0 mL of DMF. The desired amide bond was provided after 20 min reaction time in a vial at room temperature, releasing CO_2_ as the only by‐product. The reaction mixture was passed through an amino‐functionalized resin, QuadraPure BZA, contained in a column reactor, in order to eliminate the unreacted NCA. The exiting stream underwent subsequent reductive cleavage of the CBz protecting group using an H‐Cube^®^ flow hydrogenator at 0.5 mL min^−1^ over a 10 mol% Pd/C on carbon catalyst maintained at 60 °C in 10 min. Reaction with further 1.5 equiv. of Cbz‐l‐V‐NCA under identical conditions gave Cbz‐l‐V‐l‐V‐l‐F‐OMe in 95 % yield and in 95 % purity. A library of seven dipeptides was obtained with yields between 44 and 95 % and purities generally reported to be around 95 %. Furthermore, to extend the scope of this method, some peptidomimetics were achieved. (see below, Section 3).

**Scheme 7:**
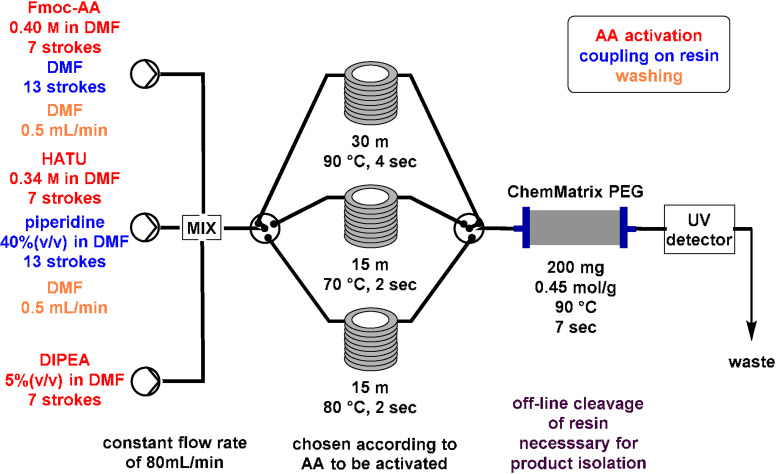
Peptide flow synthesis by Pentelute and co‐workers. Off‐line removal of peptide from resin is required after each step to prepare the starting solution for the subsequent step.^[106]^

In 2017, Pentelute and co‐workers combined the advancements of automated solid‐phase synthesis with flow chemistry elements, avoiding manual handling during the process (Table 2, entry 8; Scheme 7).^[106]^ A 0.2 m solution of Fmoc‐protected amino acid in DMF was mixed with 5 % (*v*/*v*) DIPEA and a two‐fold excess of HATU or PyBOP in DMF in the static mixer. The reaction stream was passed through a tubular reactor heated at 90 °C to trigger formation of an active ester in 2 s. The activated amino acid subsequently flowed through a packed bed of ChemMatrix polyethylene glycol (PEG) amide resin, manually preloaded at start, held at 90 °C, where amide bond formation is complete within 7 s. The authors claim that their method offers especially a time advantage, with the entire cycle for each amino acid addition being 40 s. Unfortunately, the authors did not bother to convert their set‐up‐specific “stroke”‐counting flow protocol into a more generally reproducible unit that would more directly allow manual/semi‐automatic operation of a simpler set‐up, or that facilitates general reproducibility.

A fast and sustainable flow method for the synthesis of protected peptide on solid support was developed by Szloszar et al. in the same year (Table 2, entry 9).^[107]^ They used a set‐up consisting of an HPLC pump, autosampler, column thermostat, and PEEK column.^[101]^ They synthesized the LFE‐2‐CTC‐PS sequence manually as a starting sequence for the coupling in flow. In the optimized fashion, the LFE‐TC‐Tentagel contained in the column was functionalized with 3 equiv. of Fmoc‐A‐OH, at 50° C by flowing through a solution of the protected amino acid, 3 equiv. of HATU as coupling reagent in 1.5 mL DMF, and 6 equiv. of DIPEA at 0.15 mL min^−1^ flow rate. The coupling mixture was prepared before the coupling reaction. The Fmoc group deprotection was achieved with 2 mL of 2 % (*v*/*v*) DBU and 2 % (*v*/*v*) piperidine in DMF. The peptide was cleaved from the polymer support with 20 mL of 20 % hexafluoroisopropyl alcohol (HFIP) in DCM. The peptide was achieved in an isolated yield of 94 %, with a flow rate of 0.15 mL min^–1^ in only 6.7 min. The efficiency of this method was demonstrated by the synthesis of six calcitonin and corticotropin fragments, with higher purity and shorter times than reported otherwise in literature.^[114]^

In the same year, Blacker and co‐workers developed a green and fast reaction of amino acid‐derived NCAs with unprotected amino acids under aqueous flow conditions (Table 2, entry 10).^[108]^ The water dissolved higher concentrations of free amino acids as compared to organic solvents, and the work intended to overcome problems associated with low productivity, protecting group issues, and excess NCA that reduce overall atom efficiency. This study used a custom‐made continuous stirred tank reactor with the uninterrupted automated addition of solid NCA, overflow mechanism for constant product removal, high shear mixing (4000 rpm), automated pH control, and efficient heat removal, which controlled the reaction. To the 300 mL reactor was added a 0.1 m solution of aqueous Na_2_B_4_O_7_ as buffer, and the pH was adjusted to 10.2 before cooling to 0 °C. A 0.2 m solution of aqueous amino acid was mixed with 1.1 equiv. of amino acid derived NCA, released continuously by a powder dispenser. After 7 min, the solution containing the product was collected and quenched with a solution of sulfuric acid to allow the decarboxylation of the carbamate intermediate. With this approach in hand, a library of three dipeptides and three tripeptides was obtained with conversion of 60–85 %. An additional study with automated pH controller allows to eliminate the borate buffer, a part of the process waste, holding the same productivity and efficiency. By comparing the classic SPPS method,^[115]^ the NCA in batch,^[116]^ and NCA in flow, the latter was 4600 times more productive than SPPS and 173 times more productive than NCA in batch. Furthermore, it has a 24 times better total mass intensity, mainly due to the avoidance of borate buffer, which also improves health and safety aspects.

Spare et al., in 2018, developed a manual continuous‐flow solid‐phase synthesis using a rudimentary set‐up (Table 2, entry 11).^[109]^ A pentapeptide was synthetized with two different and separate approaches: in an infusion approach, 10 mL solutions in DMF of *N*‐α‐Fmoc amino acid (0.03 m, 4 equiv.), HATU (0.03 m, 4 equiv.), and DIPEA (0.06 m, 8 equiv.) were sequentially passed at 1 or 5 mL min^−1^ through a column reactor, heated to 60 °C, and packed with a polymer‐supported Rink‐Amide (PS‐RAM) resin. In a second approach, a 1 mL DMF solution of *n*‐α‐Fmoc amino acid (0.28 m, 4 equiv.), HATU (0.28 m, 4 equiv.), and DIPEA (0.56 m, 8 equiv.) was injected into the DMF solvent stream, which passed through the resin at 1 mL min^−1^ at 60 °C. Washings were achieved with a 50 % DMF solution of piperidine that was flowed through the resin at flow rates of 5 or 10 mL min^−1^. Subsequent sequence cleavage and removal of the O*t*Bu protecting group could then be concurrently mediated with a trifluoroacetic acid (TFA)/AcOH mixture. HPLC analysis revealed overall purities of around 38 and 23 %, respectively, at 1 or 5 mL min^−1^ flow rate in the first method and <40 % in the second method, in general lower than the 90 % found for the batch process. Minimum dead space of the swollen resin surface and optimizing the flow rate and temperature to 5 mL min^−1^ and 40–60 °C, respectively, appeared optimal, affording the pentapeptide in >95 % purity. The PS‐RAM resin can be replaced by the commercially available ChemMatrix Rink amide (CM‐RAM) resin. For both resins, a 0.3 m Rink amide matrix concentration of Fmoc‐amino acid and HATU, mixed with a 0.6 m DIPEA matrix concentration at 60 °C and a flow rate of 5 mL min^−1^ gave amino acid coupling within 2.5 min. Using a 50 % solution of piperidine in DMF at 60 °C at 10 mL min^−1^, complete Fmoc‐deprotection was carried out within additional 1.5 min. This method reduced the amide bond time from 6.7 to 5 min relative to the protocol by Fülöp and co‐workers,^[101]^ while requiring more equivalents of HATU. Additionally, while 5 min is longer than the 7 s coupling period reported for the automated methodology by Pentelute and co‐workers,^[102]^ the required 8.6 equiv. of coupling reagents for CM‐RAM and 4.6 equiv. for PS‐RAM are significantly less than the 20 equiv. utilized in the fully automated protocol.

**Scheme 8:**
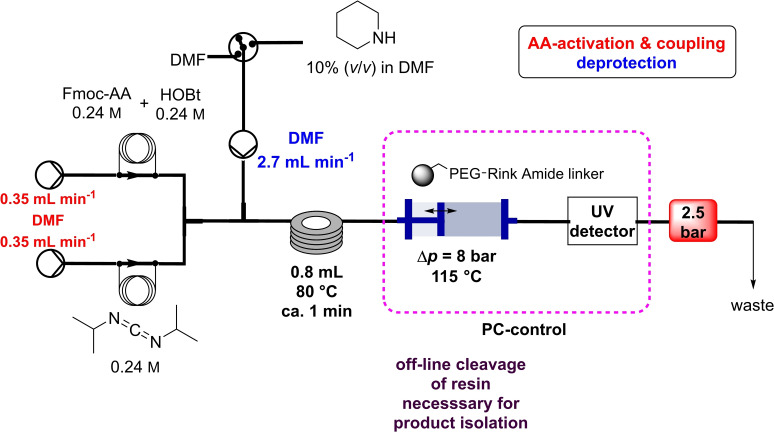
Flow synthesis to linear peptides using a variable bed flow reactor by Seeberger and co‐workers; pressure control is used to adjust bed size.^[110]^

Seeberger and co‐workers developed in 2019 an automated solid‐phase peptide synthesizer containing a variable‐bed flow reactor (VBFR) (Table 2, entry 12; Scheme 8).^[110]^ While in the fixed bed the backpressure is high and the reagent could be channeled into the bed, the variable bed maintains low backpressure and real‐time monitoring is effortless. Furthermore, the latter can automatically vary in volume to accommodate changes in volume of the packed material. The VBFR system was compatible with 4‐methylbenzhydrydrylamine (MBHA)‐RAM and TentaGel‐RAM resins. The A−F−L−A−F−L−A sequence was synthetized starting from a 0.24 m solution of Fmoc‐protected amino acid in DMF and equimolar HOBt or OxymaPure as activator, pumped by the first pump. The solution was mixed with an equimolar amount of *N*,*N*’‐diisopropylcarbodiimide (DIC), delivered by the second pump. The coupling reagents were passed at 0.7 mL min^−1^ through a heated mixing loop at 80 °C for preactivation, then into the VBFR containing the peptidyl resin at 80 °C. During acylation the resin bed volume grows; conversely, Fmoc removal contracts the resin. The overall resin bed volume increase was 0.2 mL. At the end, the Fmoc cleavage was carried out piperidine solution (10 % in DMF), flowed in by a third pump. The sequence A−F−L−A−F−L−A was achieved with around 92 % yield and 92 % HPLC purity. One synthetic cycle including two wash steps takes 16 min. To extend the scope, three biologically relevant peptide sequences (NFGAIL, FF03, TfR‐Peptide) were prepared in 73–85 % yield and 79–91 % purity. Automated VBFR‐SPPS requires only four equivalents of amino acid and is compatible with established peptide methodologies.

In 2019, Fuse et al. reported a fast and green peptide chain elongation using unprotected amino acids via mixed carbonic anhydride using micro‐flow technology (Table 2, entry 13).^[111]^ This work represents a peptide synthesis from practically unprotected amino acids or an unprotected peptide in an overall more atom‐economic and environmentally benign process. A 0.33 m solution equimolar in *O*‐benzyl‐protected dipeptide Boc‐l−A−l−S(Bzl)−OH, *N*‐methylmorpholine, and DIPEA in MeCN as green solvent was pumped by means of a syringe pump and was mixed with a 0.2 m solution of 1.2 equiv. ClCO_2_ *i*Bu in MeCN pumped by a second syringe pump, to rapidly generate mixed carbonic anhydride in situ. A solution of 1.7 equiv. of unprotected amino acid H−F−ONa in H_2_O was then added via a third stream, to carry out the coupling. In shortest times, that is, 2 s for the first reaction and 10 s for the second, the desired tripeptide was afforded in 85 % yield, with epimerization essentially suppressed to 0.4 %, as compared to the same reaction in batch, which provided 66 % yield and 0.8 % of epimerization. The scope of the methodology was additionally broadened through the substitution of different amino acids, affording a library of 14 di‐, tri‐, and tetra‐peptides with yield between 60 and 96 % and racemization rates of <1 %. Furthermore, the scale‐up potential of the flow process was demonstrated synthesizing 2.2 g of Boc‐l−A−S(Bzl)−OH without a decrease in yield.

**Scheme 9:**
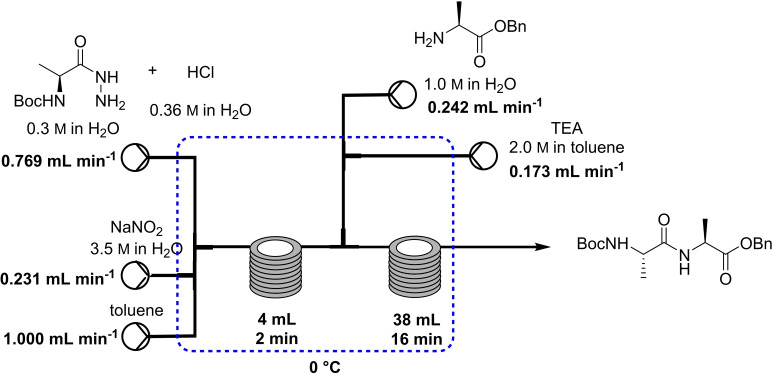
Peptide synthesis via in‐situ‐generated acyl azides as proposed by Kappe and co‐workers.^[112]^

In 2020, Kappe and co‐workers developed a new protocol for peptide bond formation that avoided side reactions, including epimerization, using acyl azide (Table 2, entry 14; Scheme 9).^[112]^ The toxic acyl azides were safely generated by using nitrous acid in water, reacting in situ within a continuous‐flow system. A solution of hydrazide substrate (4.50 mmol, 0.3 m) and 1.2 equiv. HCl (0.36 m), an aqueous 3.5 m solution of NaNO_2_, and neat toluene (batch optimization had revealed that toluene/water was best for dipeptide coupling) were pumped into a first reactor held at 0 °C for a residence time of 2 min, before mixing with, via third and fourth streams, in a 1 m aqueous solution of amino acid as HCl salt and a 2 m solution of triethylamine in toluene at 0 °C for 16 min. The small internal channel dimensions of the continuous‐flow reactor maximized the interfacial area between the aqueous and organic phases. The crude reaction mixture was collected off‐line, and the organic phase was separated. An off‐line quenching of the aqueous phase with hydrochloric acid solution eliminated any azide anions in combination with the remaining NaNO_2_. A mini‐library of 5 target compounds was synthesized, changing starting hydrazides and the amino acid, with yields between 70 and 77 %, thus being generally higher than in batch. All reactions showed very low epimerization (<1 %). A Boc‐cleavage reaction was carried out adding a 4 m HCl solution in dioxane to the *N*‐Boc‐protected dipeptide (1 mmol). The solution was mixed at 0 °C for 4 min and then at 25 °C for 10 min. The yield of five products was generally around 95 %. To explore the scope of this approach, tripeptide d−A−d−A−l−A−OBn was developed, with overall yield of 49 %.

As mentioned in the first part of this Review (see above, Table 1, entry 6), in the same year, Gordon and co‐workers developed a convenient flow protocol using polymer‐assisted carbodiimide and applied this method also to the synthesis of dipeptides (Table 2, entry 15).^[117]^ A preliminary trial employed Fmoc‐L‐OH and ethyl ester‐protected lysine in HCl‐salt form, to give the desired dipeptide in quantitative yield with no epimerization on a 0.19 mol scale. This demonstrated that HCl‐amine salts could be coupled without the inclusion of a base. A variation involving Fmoc‐(*t*Bu)S‐OH that is vulnerable to direct epimerization, and the ethyl ester protected lysine HCl‐salt revealed no epimerization using 2 equiv. of immobilized carbodiimide neither in absence, nor in presence of DIPEA, or in combination of DIPEA and HOBt.

### 2.4 Relevance of cyclic peptides and approaches to sustainable methodologies for their generation

Cyclic peptides combine several favorable properties such as good binding affinity, target selectivity, and low toxicity that make them an attractive modality for the development of therapeutics.[^118^, ^119^] Over 40 cyclic peptide drugs are currently in clinical use, and around one new cyclic peptide drug enters the market every year on average.[^120^, ^121^] Many cyclic peptides occur naturally in plants and animals,[^122^, ^123^] whereas others have been synthetically engineered to be cyclic.[^124^, ^125^] The vast majority of clinically approved cyclic peptides behave as antimicrobials and antitumor agents. New technologies associated to both the design and the in‐vitro evaluation on a variety of previously undruggable targets have prompted the de novo development of cyclic peptide ligands.

In particular, the enhanced proteolytic stability, rigidity, and ability to target protein–protein interactions make cyclic peptides appealing scaffolds for drug development.^[120]^ However, cyclic peptides remain notoriously difficult to prepare by conventional SPPS. Moreover, to separate truncated by‐products and linear sequences that failed to cyclize, extensive HPLC purification is required. Annually, 11 million L of mixed organic and aqueous waste is attributed to HPLC alone.^[126]^ Therefore, research in this area should also focus on the development of a novel chromatography‐free technology to access this privileged class of molecules.

Further challenges are associated to the development of cyclic disulfide‐rich peptides as drug candidates, which is characterized by economically and environmentally costly chemical synthesis, and low yields from both synthesis and purification.[^127^, ^128^] A more eco‐compatible and effective alternative chemical synthesis of cyclic peptide entities is the implementation of chemoenzymatic protocols, where linear precursors are synthesized using SPPS, then cyclized in vitro by native or engineered peptide ligases such as bacterial sortase,[^129^, ^130^] trypsiligase,[^131^, ^132^] subtiligase,[^133^, ^134^] or asparaginyl endopeptidases.[^135^, ^136^, ^137^] The use of peptide ligases is also applicable to large‐scale manufacturing.^[138]^ Also in this case, flow‐based approaches can represent a nice complement to both chemical and biochemical protocols to be applied to cyclic peptide synthesis. In the following paragraph, we will explore the few examples of cyclic peptide generation trough flow‐aided methodologies.

### 2.5 Synthesis of cyclic peptides in continuous flow

Fuse et al., in 2016, applied their previous triphosgene‐mediated micro‐flow amide bond formation for peptide chain elongation^[104]^ to the synthesis of a cyclic arginine‐glycine‐aspartate (RGD) peptide, behaving as a highly potent and selective antagonist for the αvβ3 integrin receptor (Table 3, entry 1).^[139]^ The development of micro‐flow photochemical macrolactamization can overcome the limit of conventionally cyclization associated with poor solubility, problematic waste originating from excess amount of coupling reagents, and harsh purification. Firstly, 5‐bromo‐7‐nitroindoline (Bni) was selected as photoactivable group and Bni‐protected Fmoc‐glycine was used as precursor of peptide chain elongation. The Fmoc cleavage of glycine was carried out in batch under basic conditions. The desired pentapeptide was yielded according to the previous procedure in flow^[104]^ (see above, Table 2, entry 6) in 8 steps with 7 % overall yield, with only one step of purification. After Boc‐deprotection with TFA in CH_2_Cl_2_ (1 : 3), a solution of pentapeptide in MeCN was introduced into the tube reactor at room temperature with a syringe pump at 600 μL min^−1^ and irradiated with a 9 W UV lamp at a wavelength of 365 nm for 5 min. The cyclic pentapeptide was obtained in 36 % yield and the subsequent hydrogenation furnished the desired cyclic RGD peptide in quantitative yield and <95 % purity by NMR spectroscopy.

**Table 3:** Formation of cyclic peptides in continuous flow.

Entry	Starting compound(s)	Coupling reagent(s)/ catalysts	Targeted structural scope	Solvent./*T*	Reactor	# Examples/ yields [%]	Ref.
1	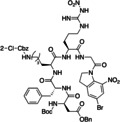	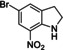	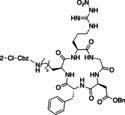	MeCN 25 °C	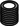	1 36	[139]
		*hν*					
2	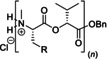	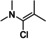	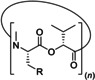	DCM 25 °C	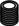	6 32–52	[140]
		DIPEA					
3	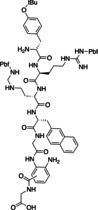	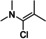	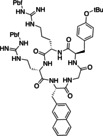	DMF 25 °C	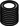	1 80	[141]
		DIPEA					
4	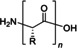	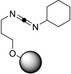	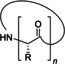	DCM–DMF 60 °C	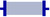	9 >95^[a]^	[73]

[a] Only conversions reported.

**Scheme 10:**
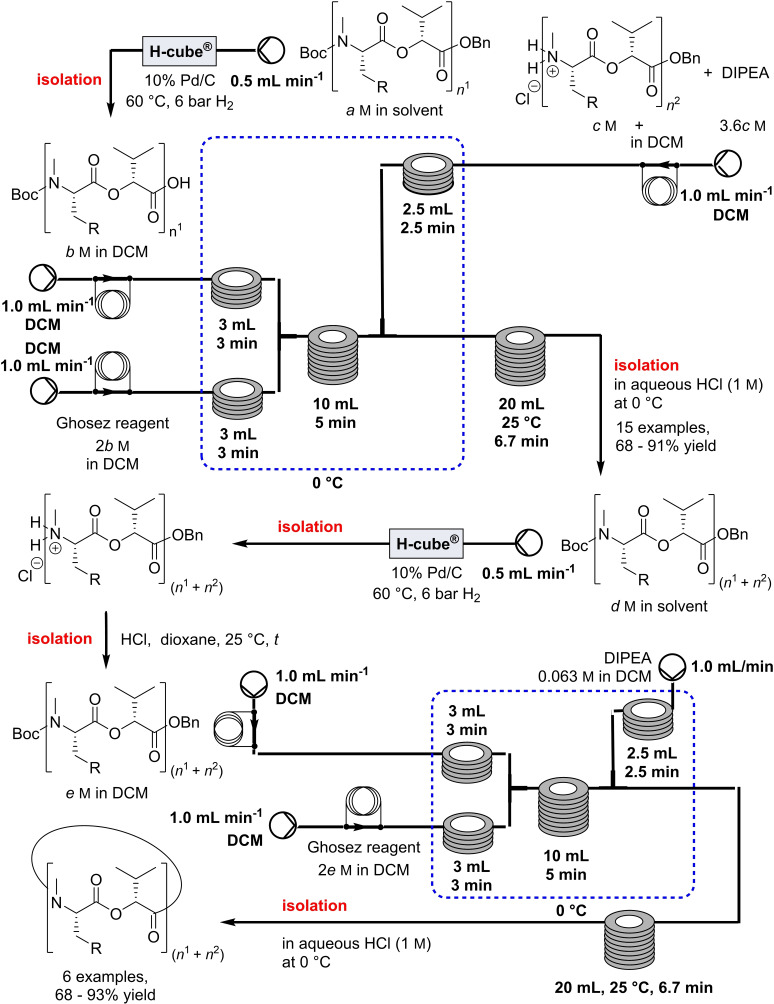
Flow synthesis of cyclic peptides proposed by Lücke et al.^[140]^

In the same year, Lücke et al. developed a protocol for the synthesis of cyclooligomeric depsipeptides (CODs) that exhibit a wide variety of biological activity, including antibiotics or insecticides (Table 3, entry 2; Scheme 10).^[140]^ The previous synthesis of these compounds was problematic in terms of yields of 2–9 % and costs.[^142^, ^143^, ^144^]

The fully optimized semi‐telescoped flow‐approach provided the desired six compounds with yields between 32 and 52 %, being all higher than the ones obtained for the same reaction in batch. The protected building blocks (0.05 mmol) were synthetized in batch in 14.5 h in semi‐quantitative yield. The employed reactor was equipped with three inlets, and plunger pumps were used to introduce solutions into the reactor via these inlets after passage through a precooling unit. The crude acid, obtained in a preceding reductive deprotection of the benzyl‐protected precursor in an H‐cube over a 10 % Pd/C catalyst, was mixed in the reactor with a solution of 2 equiv. of Ghosez reagent in DMC at 1 mL min^−1^ per pump to activate the carboxylic acid as acid chloride for 5 min at 0 °C. The crude amine HCl salt, obtained in batch in a classic HCl‐mediated Boc‐deprotection protocol, and a 3.6‐fold excess diisopropylethylamine in DCM were then added via third stream through the third inlet at 1 mL min^−1^ and mixed with the first coupling partner for approximately 7 min. Once the third stream had been added, the coupling reaction could be allowed to proceed at room temperature. The reaction outflow was collected in 1 m aqueous HCl at 0 °C, affording the crude linear product. With the linear precursor in hand, further deprotection steps removed both the Boc and Bn groups. Activating the acid as described above, and adding diisopropylethylamine, 0.036 m in DCM, allows the intramolecular macrocyclization. Six cyclooligomeric depsipeptides were obtained with yield between 68–93 %.

**Scheme 11:**
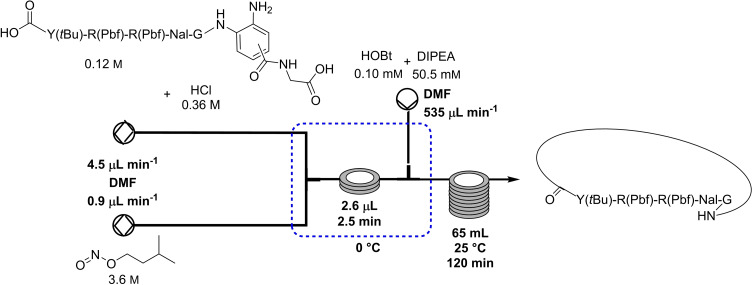
Micro‐flow synthesis of cyclic peptides proposed by Oishi and co‐workers.^[141]^

In 2018, Oishi and co‐workers reported the micro‐flow synthesis of a cyclic peptide via 3,4‐diaminobenzoic acid (Dbz) as active ester precursor (Table 3, entry 3; Scheme 11).^[141]^ The solvent for both reactants was DMF. A 120 mm solution of d‐Y(*t*Bu)‐R(Pbf)‐R(Pbf)‐Nal‐G‐(Dbz), containing the Dbz‐linker and 3‐fold excess of HCl was pumped by means of a syringe pump at a rate of 4.5 μL min^−1^ and mixed with a 3.6 m solution of isoamyl nitrite at 0.9 μL min^−1^, at 0 °C for 2.5 min. A 50.5 mm solution of DIPEA containing 0.101 mm HOBt was introduced into the reactor via syringe pump at 534.6 μL min^−1^, via third stream, to produce the desired cyclized product in 2 h at room temperature. The reaction was quenched off‐line with aqueous HCl solution, and the protected cyclic peptide was obtained in satisfactory yield of 80 % and good purity by NMR spectroscopy.

In their publication of 2020 (see above, Table 1, entry 6; Table 2, entry 15), Gordon and co‐workers developed a convenient flow protocol using polymer‐assisted carbodiimide and applied this method also to cyclic peptides (Table 3, entry 4).^[117]^ The required linear sequences were synthesized using a previously reported continuous‐flow peptide coupling protocol with side‐chain protecting groups maintained.^[109]^ A solution of a linear peptide, 0.02 m in DCM with 10 % DMF, was injected into the continuous DCM stream pumped at 0.5 mL min^−1^ through a column packed with 2 equiv. of carbodiimide‐functionalized resin held at 60 °C. A library of five cyclic dipeptides and four cyclic pentapeptides was obtained in >95 % conversion and >95 % yield.

### 2.6 Peptidomimetics: relevance and efforts towards sustainable protocols for their generation

In parallel to the growing prominence of the peptidomimetic drug design approach in various fields of pharmaceutical chemistry and drug design in general, the synthetic methodologies applied to realize diverse peptidomimetic substructures have been increasingly improved.[^145^, ^146^] However, the several approaches introduced so far for their generation suffer from high number of steps and poor overall efficiency.[^147^, ^148^, ^149^, ^150^, ^151^]

Standard SPPS protocols have also been modified in order to adapt to the generation of peptidomimetic scaffolds. Accordingly, they were combined with click chemistry protocols, metal‐mediated reactions, *N*‐alkylations, and *N*‐acylations in order to incorporate diverse building blocks and functionalities.^[152]^ As for traditional SPPS, one of the future development directions for the peptidomimetic solid‐phase synthesis will be the incorporation of green solvents and recyclable solid supports.

Isocyanide‐based multicomponent reactions (MCRs), like the Ugi reaction and its variants, also increasingly became useful tools for creating novel peptidomimetic structures, owing to the fact that diamide “peptoid” motifs are inherently created in the course of these reactions,[^153^, ^154^, ^155^, ^156^] with a reduced numbers of synthetic steps and, in general, improved sustainability features.[^157^, ^158^, ^159^, ^160^] Particularly attractive is the opportunity to construct, via a MCR, (spiro)cyclic, constrained peptidomimetics, which are expected to possess improved absorption properties due to reduced number of rotatable chemical bonds and to better mimic characteristic structural biases in peptides and proteins, such as beta‐turns.[^161^, ^162^, ^163^] Recent steps towards efficient peptidomimetic synthesis were made by coupling biocatalysis to MCRs, through selective laccase‐oxidation of alcohol to a corresponding aldehyde and a following Ugi reaction.^[164]^

Of course, in this frame, the synergy with flow‐based approaches represents an appealing horizon, still at its initial stages of exploration for peptidomimetic synthesis. The most significant examples in this context are reported in the next paragraph.

### 2.7 Synthesis of peptidomimetics in continuous flow

As already mentioned in the previous sections (Table 2, entry 6), in 2014, Fuse et al. developed in their group a flow route to achieve peptide couplings that could be used also for generation of peptidomimetics, starting from various amino acids and triphosgene as activator (Table 4, entry 1).^[104]^ Applying the conditions optimized for the amide bond formation, two peptidomimetic compounds were synthetized. Compared to the equivalent batch syntheses, the flow protocol improved the space‐time yield from 53 and 28 %, respectively, to 74 and 98 %, with epimerization of <1 %.

**Table 4:** Peptidomimetics in flow.

Entry	Starting compound(s)^[a]^	Coupling reagent(s)/ catalysts	Targeted structural scope	Solvent/*T*	Reactor	# Examples/ yields [%]	Ref.
1	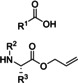	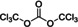	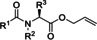	DMF–MeCN 20 °C	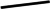	dimers: 2 74–98	[104]
		DIPEA					
2	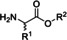	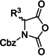	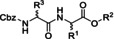	DMF 25 °C	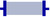	trimers: 4 44–53 pentamers: 1 32	[105]
					H‐cube		
3^a^	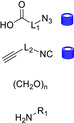	–	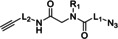	MeCN–MeOH 80 °C	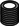	8 80–95	[165]
4	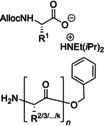	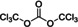	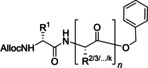	DMF–MeCN–H_2_O 20 °C	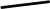	4 70–83	[166]
		DMA					
5	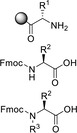	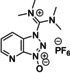 DIEA	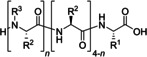	DMF 60 °C	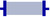	8 92–95	[167]
6	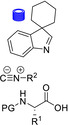	–	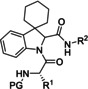	EtOH 80 °C	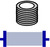	4 50–55	[168]

[a] Small blue reactor symbols close to substances indicate their in‐situ generation during the protocol.

Ley and co‐workers, in their already mentioned paper from 2014 (Table 2, entry 7),^[105]^ incorporated also unnatural amino acids into the reaction sequence (Table 4, entry 2). With the optimized reaction conditions in hand, Cbz‐d‐α‐cyclohexyl‐G‐NCA reacts with d−P−O*t*Bu and Cbz−d−α‐(*p*‐fluorophenyl)−G−NCA to form a tripeptide comprised of two amino acid mimetics and one unnatural amino acid in 52 % isolated yield and 80 % purity. Furthermore, Cbz‐d−α‐(*p*‐fluorophenyl)−G−d‐α‐Cyclohexyl‐G−lvF−l−L−l−V−d−P−O*t*Bu is readily formed in 32 % isolated yield and 75 % purity.

**Scheme 12:**
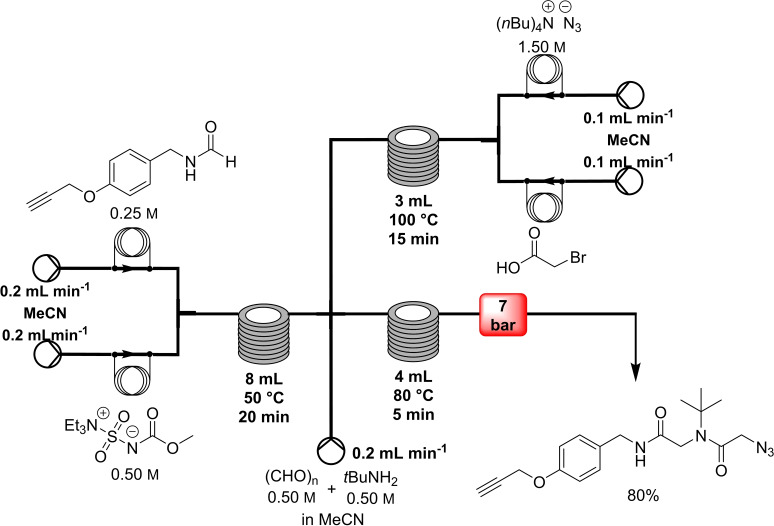
Flow synthesis of linear peptoids via Ugi four‐component reaction using in‐situ generated reagents by Kappe and co‐workers.^[165]^

In 2015, Kappe and co‐workers reported the synthesis of linear peptoids via isocyanide‐based MCR and their subsequent macrocyclization via click chemistry (Table 4, entry 3; Scheme 12).^[165]^ Peptoids, or poly‐*N*‐substituted glycines, are a class of peptidomimetics whose side chains are annexed to the nitrogen atom of the peptide backbone, rather than to the α‐carbons. These structures present numerous advantages include ease synthesis, higher proteolytic stability, and bioavailability compared to the natural peptides. A flow Ugi four‐component reaction (U‐4CR) between amine, oxo building blocks, isocyanide, and azide furnished the corresponding linear peptoid. Since the isocyanide is highly reactive, apart from the characteristic unpleasant smell, an upstream dehydration step of the respective formamide can bypass these problems, developing the isocyanide in situ. In a similar fashion, the hazardous azide building blocks were prepared via an upstream protocol from a bromide precursor. In a telescoped approach, a 0.25 m solution of formamide in MeCN was pumped and mixed with Burgess reagent (0.5 m in MeCN) at an overall flow rate of 400 μLmin^−1^ for the isocyanide formation. After passing in a coil reactor held at 50 °C over 20 min, the stream was directly mixed with the outcome of the azide that was obtained after 7 min by a reaction between a 1 m bromide solution and a 1.5 m solution of *tert*‐butyl acetoacetate (TBAA) in MeCN at 100 °C, and with a 0.5 m solution of paraformaldehyde and *tert*‐butylamine in MeOH. The output stream, at an overall flow rate of 0.8 mL min^−1^, was then heated to 80 °C for 5 min to achieve the U‐4CR reaction. A library of 8 linear peptoids was obtained in only 25 min, with no intermediate purification step and with increased safety, in yields between 80 and 95 % after a concluding off‐line purification step. Subsequent macrocyclization via copper‐catalyzed 1,3‐dipolar cycloadditions resulted in the 1,4‐substituted 1,2,3‐triazole scaffold. The 1,2,3‐triazole scaffold gave rigidity to the structure and mimed the amide bond in either its *cis*‐ or *trans*‐like configuration. A 2 mm solution of the respective linear peptoid in equivolume MeCN/MeOH was pumped at 800 μL min^−1^ through a copper coil reactor heated at 140 °C in a gas chromatography (GC) oven for triggering final cyclization. After purification via preparative HPLC, 8 cyclic peptoids in analytical purity with yields between 71 and 91 % were obtained.

In 2016, Fuse et al. applied their previous work^[104]^ (Table 2, entry 6) to the total synthesis of feglymycin, a biologically active oligopeptide composed of thirteen amino acids involving 4‐hydroxyphenylglycine (Hpg) and 3,5‐dihydroxyphenylglycine (Dpg) that is very prone to racemization (Table 4, entry 4).^[166]^ Hpg and Dpg are the most important aryl glycines, being important non‐proteinogenic amino acids. Thanks to the reduction of residence time, the racemization was suppressed (≈3 %), and only CO_2_ and HCl salt of DIPEA was produced as byproduct. Feglymicine, a tridecapepide, was synthetized in a convergent approach using a heptameric and a hexameric pseudopeptidic building block, each produced in turn with the presented method via peptide chain elongation.

A fast and sustainable continuous‐flow solid‐phase peptide synthesis protocol of mono‐ and multiple‐*N*‐methylated peptides was developed by Fülöp and co‐workers in 2018,^[167]^ relying on their previous work (Table 4, entry 5).^[101]^ A mixture of 1.5 equiv. of Fmoc‐protected amino acid, equimolar amounts of HATU as coupling reagent, and two‐fold excess of DIPEA in 1 mL DMF was passed at 0.15 mL min^−1^ flow rate through a column packed with Tentagel resin‐bound alanine or valine tetrapeptide and heated to 60 °C. For Fmoc deprotection, 2 mL of a DMF solution containing 2 % (*w*/*w*) DBU and 2 % (*w*/*w*) piperidine has been used. According to the best conditions, 4 *N*‐methylated oligoalanines were synthesized in yields greater than 92 %. Since coupling efficiencies were acceptable for alanine peptides, the technology was tested on difficult sequences, including oligovalines, that could be obtained in high purities, adjusting the conditions for each reaction.

Furthermore, the scale‐up potential of the flow process was demonstrated synthesizing 0.15 mmol scale of *N*‐methyl‐*N*‐(*N*‐methyl‐*N*‐(methyl‐d−A)−l−A)−l−A−l−A−l−A with 93 % yield. With this approach, the coupling and deprotection were carried out in 28 min under flow conditions, compared to approximately 3 h in the literature‐reported batch protocol, using only 4.2 mL of solvent compared to 66 mL reported in the literature for the batch process, and avoiding three additional chemical steps. The same compound was subjected to solution phase cyclization. A solution of 1 equiv. of *n*‐methyl‐*N*‐(*N*‐methyl‐*N*‐(methyl‐d−A)−l−A)−l−A−l−A−l−A in 10 mL DMF and a 1.5 equiv. of HATU in 10 mL DMF were flowed by means of a syringe pump at flow rate of 0.015 mL min^−1^ into a stirred solution of 3 equiv. of DIPEA in 40 mL DMF, for a total reaction time of 30 min. The conversion was quantitative and the purification using reversed‐phase HPLC provided 68 % isolated product liberated from resin for pseudopeptide (3*S*,6*R*,9*S*,12*S*,15*S*)‐1,3,4,6,7,9,12,15‐octamethyl‐1,4,7,10,13‐pentaazacyclopentadecane‐2,5,8,11,14‐pentaone; this yield is three times higher than the published 26 % for the batch process.^[169]^

**Scheme 13:**
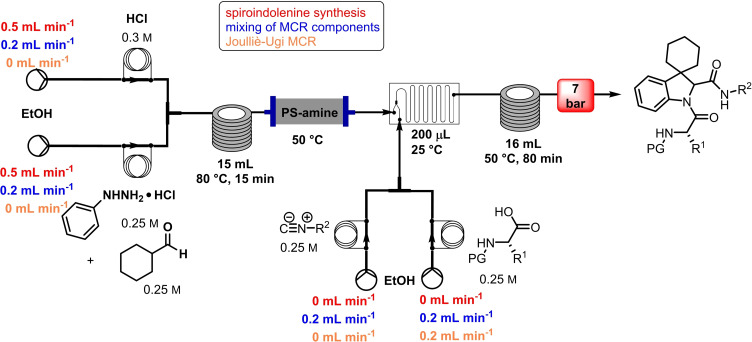
Ugi–Joulliè‐mediated synthesis of peptidomimetics proposed by Alfano et al.^[168]^

One of the latest works was recently published by Alfano et al. They reported a green and sustainable diversity‐oriented synthesis of a library of peptidomimetics through Ugi–Joulliè multicomponent reactions (Table 4, entry 6; Scheme 13).^[168]^ Having developed in a previous work a continuous‐flow synthesis of 3,3‐disubstituted indolenine through interrupted Fischer indolization reaction,^[170]^ they used spiroindolenines as substrate for the generation of peptidomimetic frameworks. A 0.25 mm solution of equimolar amounts of spiroindolenine, suitable amino acid, and isocyanide was injected in a flow system comprised of a 15 mL PTFE coil reactor heated to 80 °C, using a total flow rate of 0.2 mL min^−1^ for achieving a residence time of 75 min. Compared to the results of a preliminary batch investigation, transition to flow reduced time need with respect to batch synthesis of 95 %. Furthermore, based on their previous work, a telescoped approach coupling the initial interrupted Fischer reaction for spiroindolenine synthesis with the subsequent Joulliè–Ugi‐type modification was established as well. Using only approximately 15 % of the combined amount of solvent that was necessary for the two batch processes including purifications, and only 100 min for the entire sequence in flow instead of 24–48 h for the batch mode, four different peptidomimetics were realized in very competitive yields of around 50 %.

## 3 Conclusions and Outlook

Although the first paper reporting a flow‐based synthesis of polypeptides was reported in 2006, it took nearly another decade before the flow chemical approach took off in peptide synthesis. Since 2014, a notable increase in reports can be found, seeing alongside the mere transformation of working batch processes without innovative changes into flow also some interesting works in which novel flow reactors were introduced to account for some of the peculiarities of peptide synthesis in flow or on solid support.^[110]^ Most of the works aim at an improvement in space‐time yields, allowing for generation of peptide bonds within seconds in extreme cases on smallest scales.[^102^, ^106^] Rendering the synthesis of amide bonds and/or oligo‐ or polypeptides more sustainable, or green, or eventually both, seems yet to have been of secondary importance. Rather large excesses of reagents and tremendous atom economies due to traditional protecting group games are often encountered also when performing peptide synthesis in flow. The use of polymer‐supported reagents, or polymer supports for the amide to be synthesized, enables obviously the flow process, but represents nevertheless eventually a burden in terms of overall process economy in case it is not possible to recycle and reuse the polymer supports. The challenges for future works in this area are thus sketched already. It might be worthwhile to look for exploiting the possibilities offered by the combination of flow synthesis and synthesis on support to think of an adaption of existing protecting group strategies and/or protecting groups, or, even more desirable, for fully abolishing the need of protecting groups. Only three approaches so far use the possibilities of increased reaction control in flow for realizing essentially protecting group‐free syntheses.[^102^, ^103^, ^108^, ^113^] An equally small number of papers, just two, use the possibility in flow to generate instable starting materials needed for the formation of the amide bond motif in situ and/or in a telescoped manner.[^165^, ^168^] Interestingly, no reports exist yet that use enzymes in flow processes for amide bond formation without the need of protecting groups. While this can be understood given the peculiarities connected to using enzymes in synthesis, it is even more interesting that approaches using photochemistry and metal–organic reagents have been only scarcely applied in flow,^[70]^ despite successful outcomes in batch amide synthesis (see above) and despite the fact that these types of chemistry have been successfully applied as such in flow mode.^[45]^ In terms of removing potentially necessary excesses of reagents, surprisingly, efficient in‐line purifications have not been widely reported yet. Also, yet another point for further improvements are the solvents used. Only very few works use environmentally benign solvents. *N*,*N*‐dimethylformamide (DMF) is still the solvent of choice, but also chlorinated solvents are still found even in more recent efforts. Here, at least, a reduction of the solvent needed overall can be seen as the step towards an environmentally more benign synthesis.

The observations made regarding the green chemistry and sustainability aspects can maybe be explained by the fact that, in the biomedical sector, for a series of good reasons, space‐time yields and synthetic reliability are the all‐determining key aspects, making environmental concerns come second. Given the pressing needs faced by modern societies in the latter aspect, however, it might be time to start looking for a working compromise between a satisfying performance and an environmentally acceptable synthetic approach a bit more seriously.

We hope that this Review will help turn the spotlight on sustainability issues in flow‐aided amide bond generation and foster further research efforts in the field.

## Conflict of interest

The authors declare no conflict of interest.

## Biographical Information

*Antonella Ilenia Alfano is currently pursuing her Ph.D. in the SPOTS‐Lab at the Department of Pharmacy at the University of Naples ‘Federico II’. She graduated in 2018 in Pharmaceutical Chemistry and Technology. Soon after she spent six‐month in a pharmaceutical chemistry laboratory working on the synthesis of active compounds via visible‐light photocatalysis approaches. Her Ph.D. project focuses on the development of greener and more sustainable syntheses of privileged scaffolds through the use of continuous‐flow techniques. Moreover, she is working on flow chemistry applications for the synthesis of diversity‐oriented libraries of small molecules and nature‐inspired compounds*.

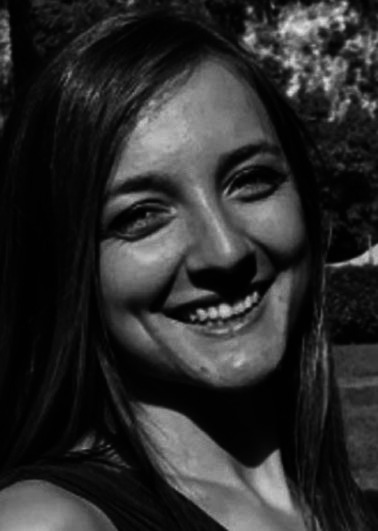

## Biographical Information

*Heiko Lange obtained his Ph.D. in Organic Chemistry from the Westfälische Wilhelms‐Universität Münster in 2008. He conducted post‐doctoral research with Prof. Steven V. Ley at the University of Cambridge, UK, with Prof. G. M. Whitesides at Harvard University, and with Prof. C. Crestini at the University of Rome ‘Tor Vergata’, where he also started his independent academic career as Assistant Professor before moving to the University of Naples ‘Federico II’. In 2021 he was appointed Associate Professor for General and Inorganic Chemistry at the Department of Earth and Environmental Sciences at the University of Milano‐Bicocca. His research interests comprise sustainable and/or green syntheses and the exploitation of natural polyphenols for pharmaceutical and material science applications*.

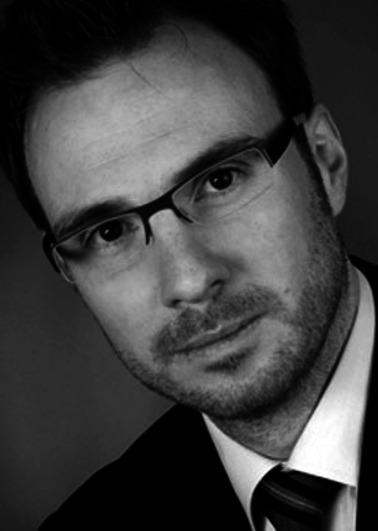

## Biographical Information

*Margherita Brindisi received her Ph.D. in Pharmaceutical Sciences from the University of Siena (Italy) in 2008. As a temporary researcher in Siena, she worked on developing compounds for the treatment of cancer, viral and parasitic diseases, and rare disorders. She was a postdoctoral fellow in Professor Arun K. Ghosh's research group in the Department of Chemistry at Purdue University (USA) in 2010–2011 and a Visiting Scientist in the same group in 2016–2017. In April 2019, she was appointed as Assistant Professor at the Department of Pharmacy at University of Naples ‘Federico II’. Margherita is currently PI at SPOTS‐Lab, where she is working on the application of sustainable methodologies to her drug discovery projects, with a particular focus on peptidomimetics and heterocyclic scaffolds*.

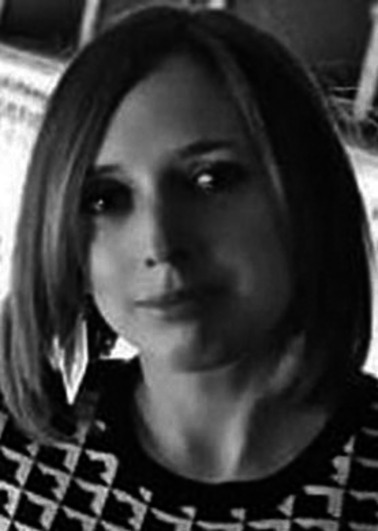
